# Comparison and Analysis of Gut Microbiota in Children With IgA Vasculitis With Different Clinical Symptoms

**DOI:** 10.3389/fped.2021.800677

**Published:** 2022-01-07

**Authors:** Meng Li, Xiaoming Wang, Xingjie Lin, Xiuju Bian, Rui Jing, Andrew Frelinger, Aijun Zhang

**Affiliations:** ^1^Department of Pediatrics, Qilu Hospital of Shandong University, Jinan, China; ^2^Department of Pediatrics, The Fifth People's Hospital of Jinan, Jinan, China; ^3^Department of Pediatrics, The People's Hospital of Weifang, Weifang, China; ^4^Dana-Farber/Boston Children's Cancer and Blood Disorders Center, Harvard Medical School, Boston, MA, United States

**Keywords:** children, gut microbiota, sequencing, immune disease, IgA vasculitis

## Abstract

**Background:** Henoch-Schönlein purpura, now called immunoglobulin A (IgA) vasculitis, is a common autoimmune disease in children, its association with gut microbiota composition remains unknown.

**Methods:** The collected cases were divided into three groups: G1 group of simple skin type, G2 group with no digestive tract expression, G3 group of mixed digestive tract, and C group of healthy children. The fecal samples of each group of children were collected and the sequencing data was processed and analyzed. The dilution curve reflected the reasonableness of the amount of sequencing data.

**Results:** The number of species composition sequences in the G1, G2 and G3 groups was lower than that in the C group, especially for the G2 and G3 groups. The four most abundant bacteria were Bacteroidetes, Firmicutes, Proteobacteria and Actinobacteria. The relative abundance of Proteobacteria in the G2 and G3 groups was significantly higher than that in the G1 and C groups, while the relative abundance of Actinobacteria was significantly reduced, and the relative abundance of Actinobacteria in the G1 group was lower than that in the C group. Principal component analysis of the UPGMA clustering tree and each group of samples showed that the microbial community composition of the same group of samples was similar.

**Conclusions:** The abundance of intestinal microbes in children with IgA vasculitis is lower than in normal children. Bacteroidetes, Firmicutes, Proteobacteria and Actinobacteria are the four most abundant bacteria in the intestinal flora of children. Proteobacteria and Actinobacteria are associated with organ involvement in IgA vasculitis.

## Introduction

Immunoglobulin A (IgA) vasculitis, is a systemic vasculitis that is common in pediatrics. It occurs mainly in children between 3 and 10 years of age. Children with mild conditions have only purpura, and some with severe symptoms which could have multiple affected systemic organs, including skin, gastrointestinal tract, joints and kidneys. The incidence of IgA vasculitis with mixed syndromes has been increasing recently (1). The long-term or repeated use of steroids and immune-suppressants, especially for children with gastrointestinal and renal involvement, can easily lead to infection, electrolyte imbalance, and a series of complications that seriously affect their quality of life (2). Therefore, how to effectively prevent and reduce the occurrence of allergic purpura involving vital organs in children is very important.

The development of the metagenomics program has led to the discovery of the interrelationships and effects of the gut with its microbial flora, and providing how different community compositions affect various states of human health at all ages, from infancy to old age (3). We hypothesized that children with IgA vasculitis who have intestinal involvement would have altered fecal microbial flora than the ones without intestinal involvement. Moreover, whether IgA vasculitis patients without intestinal involvement would have intestinal flora that was not significantly different from that of healthy controls is still unknown. To explore the intestinal flora differences of children with IgA vasculitis involving different organs, the study aimed to establish a clearer picture of the differences among children with different types of IgA vasculitis, and identify whether healthy fecal microbiota could potentially be used for the clinical treatment of IgA vasculitis.

## Materials and Methods

### Patients

#### IgA Vasculitis Patients With G1-G3 Group

The G1-G3 group was composed of children with IgA vasculitis. They were selected from the hospitalized children at Department of Pediatrics of Qilu Hospital of Shandong University from Octobor 2017 to September 2019.

G1 patients are accord with the criteria as the following: (a) clinical symptoms and physical signs consistent with the diagnostic criteria (4) for IgA vasculitis; (b) no abnormality of the joints, gastrointestinal tract, or urine detecting during examination; (c) in the acute stage of the disease, systemic treatment including the application of corticosteroids has not been started yet; and (d) no antibiotics or microecological preparations have been used in the past month. Fecal samples from children with IgA vasculitis and meeting this enrolment criteria (*n* = 20) were collected in sterile centrifuge tubes and stored at −78°C. Notably, 15 of the 20 subjects had other symptoms of IgA vasculitis within 6 months after being selected.

G2-G3 patients are cellected according to the following criteria: (a) clinical symptoms and physical signs consistent with the diagnostic criteria for IgA vasculitis; (b) presentation of joint or gastrointestinal symptoms or signs or of abnormal urine; (c) in the acute stage of the disease, systemic treatment has not been started yet; (d) exclusion of other possible factors that can lead to joint, gastrointestinal, or urinary abnormalities; and (e) no antibiotics or microecological preparations have been used in the past month. IgA vasculitis patients meeting this criteria were divided into two groups: those without gastrointestinal symptoms, G2 group (*n* = 15), and those with gastrointestinal symptoms, G3 group (*n* = 12). Fecal samples were collected and stored by the methods described above.

#### Healthy Control Group

The healthy control group (CON) included 15 healthy children who were randomly selected from children's health clinics according to the following criteria: (a) no diarrhea or other gastrointestinal diseases within the previous 4 weeks; (b) no antibiotics or microecological preparations have been used in the past month; and (c) normal results from a routine examination of the stool. Fecal samples were collected and stored by the methods described above.

## Methods

The methods are similar to the previously described method (5, 6), and the modification is as following.

### Extraction of Genome DNA

The total genomic DNA was extracted from the sample using the CTAB/SDS (7) method. The concentration and purity of DNA were monitored by separation on a 1% agarose geland diluted to a concentration of 1 ng/μl with sterile water.

### Amplicon Generation

The diluted genomic DNA was used as a template; specific primers with Barcode were used according to the selection of the sequencing region; Phusion® High-Fidelity PCR Master Mix with GC Buffer was used. The PCR was performed using efficient and high fidelity enzymes to ensure amplification efficiency and accuracy. Primer corresponding area: 18S V4 area-528F-706R; 16S V4 area-515F-806R; ITS1 area-ITS1F-ITS2; ITS2 area-ITS2-3F-ITS2-4R; 18S V9 area-1380F-1510R.

### PCR Product Mixing and Purification

The samples were mixed at the same concentration according to the concentration of the PCR product, thoroughly mixed, and the PCR product was detected by 2% agarose gel electrophoresis, and GeneJET gel (Thermo Scientific) was used. The product was recovered.

### Library Preparation and Sequencing

The library was constructed using the NEB Next® UltraTM DNA Library Prep Kit for Illumina Library. Qubit quantification and library detection were performed on the constructed library. After passing the test, the MiSeq was used for sequencing on the machine.

## Data Analysis

### Sequencing Data Processing

The raw data obtained by the Illumina MiSeq/HiSeq sequencing platform has some low quality data that will interfere with the final result. Therefore, it is necessary to preprocess the offline data before further analysis. The specific processing steps are as follows: Data splitting, PE Reads stitching, Tags filter and Tags to chimera sequences. The PE Reads splicing is performed with the application of FLASH (V1.2.7, http://ccb.jhu.edu/software/FLASH/) (8) to split the data for the reading of each sample. Raw Tags are also the stitching sequences obtained.

### OUT Cluster and Species Annotation

All of the Effective Tags sequences of all samples were clustered using Uparse software (Uparse v7.0.1001, http://drive5.com/uparse/) (9), providing clustering with 97% and 95% consensus sequences to become OTUs results, whichpurpose is to study the compositional diversity information of the species of the sample. A sequence in the same OTU is considered to be a sequence derived from one of the same taxon as the hypothetical taxon. When Uparse constructs OTUs, it selects the sequence with the highest frequency according to its algorithm principle, and uses these RDP Classifier and GreenGene database for species annotation analysis to study the phylogenetic relationship between OTUs and uses KRONA for species identification. The results of the annotations are visualized. Based on the species annotation, the number of sequences for each sample at each classification level is calculated, and the sequence of species constitutes a histogram.

To facilitate further study of the phylogenetic relationships of OTUs and the structural differences of major flora between different samples (groups), phylogenetic relationship data for the first 10 genera of OTUs corresponding to the maximum relative abundance were selected and combined with each OTUs. Relative abundance and species annotation confidence for representative sequences, the results of the integration can visualize the diversity of the species composition of the study. According to the type labeling and abundance information of all samples of the genus level, select the top 35 abundance genus and its abundance information in each sample to draw a heat map, and collect clusters from the difference between the classification information and the sample to identify Focus on more species or samples in the study sample. Select the phylogenetic relationship data of the OTUs corresponding to the top 10 relatives of the largest relative abundance and the relative abundance information of their corresponding OTUs to achieve vertical clustering of samples at the OTUs level to examine the differences between different samples or Similarity.

### Alpha Diversity

Alpha Diversity is used to analyze community diversity within a sample and includes three indicators: dilution curve, species richness, and community diversity. The sample complexity index was calculated and plotted using Qiime software (Version 1.7.0). The Rarefaction Curve is used to indicate whether the amount of sequencing data of the sample is reasonable and indirectly reflects the richness of the substance in the sample. It is a curve obtained by randomly extracting a certain amount of sequencing data from a sample to calculate the number of species they represent, based on the number of species and the amount of data. In the dilution curve, when the curve tends to be flat, it means that more data will only produce a small amount of new OTU, indicating that the amount of sequencing data is reasonable.

### Beta Diversity

Principal Component Analysis (PCA) is a method for dimensionality reduction of multidimensional data and the most important elements and structures in the data by applying variance decomposition (10). It was applied to reduce the dimension of the original variables using the QIIME software package (V1.7.0, http://qiime.org/index.html) (11). It can reflect the difference of multi-dimensional data on the two-dimensional coordinate map, and the method of selecting the two coordinate axes that can reflect the difference between samples is selected from the PCA results. The closer the sample is in the PCA plot, the more similar its community composition is. UPGMA (Unweighted Pair-group Method with Arithmetic Mean) is a commonly used cluster analysis method in environmental biology. It requires a transformation from the distance matrix to a new set of orthogonal axes, where the maximum variation factor is represented by the first principal coordinate, the second maximum is represented by the second primary coordinate, and so on (12). UPGMA clustering is a hierarchical clustering method that uses average links and can be used to interpret the distance matrix (13).

### Statistical Processing

The measurement data were expressed as mean ± standard deviation (x ± s), the rank data were analyzed by rank sum test, and analyzed by SPSS 20.0 software. The mean comparison between the two groups was performed by *t*-test, and the mean of the count data was compared. The Dunnett-t test was used for multi-sample multiple comparison. The Wilcoxon rank sum test was used for grade data. The overall rate was compared by χ2 test. *P* < 0.05 was considered statistically significant. Differences in individual abundance between the two groups were confirmed using Metastats software (http://metastats.cbcb.umd.edu/) (14, 15). LEfSe (http://huttenhower.sph.harvard.edu/galaxy) was used for quantitative analysis of biomarkers in different groups. The purpose of this method is to analyze data on the number of species much higher than the number of samples to determine the statistical significance, bio-identity and effect size estimates predicted biomarkers (16, 17). In order to determine the difference in microbial communities between the two groups, ANOSIM (18) and MRPP (multi-response permutation procedure) (19) were performed based on the Bray-Curtis distinct distance matrix.

## Results

### Patient Characteristics

Firstly, 20 children were selected for the G1 group, but 15 of the 20 subjects had other symptoms of IgA vasculitis within 6 months after being selected. Therefore, only 15 samples were included in the statistical analysis of the G1 group. The average ages of the G1 (*n* = 15), G2 (*n* = 15), G3 (*n* = 12), and healthy controls (C group, *n* = 15) were 5.1 ± 1.3 y, 5.4 ± 0.9 y, 6.3 ± 1.1 y, and 4.9 ± 0.7 y, respectively. The ratio of male to female is 1:1.14, 1:1.50, 1:0.71, and 1:0.88. There were no significant differences in age and gender among the three groups (*t* = 1.42, *p* > 0.05).

### Intestinal Microbiota Diversity Among Children With Different Types of IgA Vasculitis

**Figure 1:**
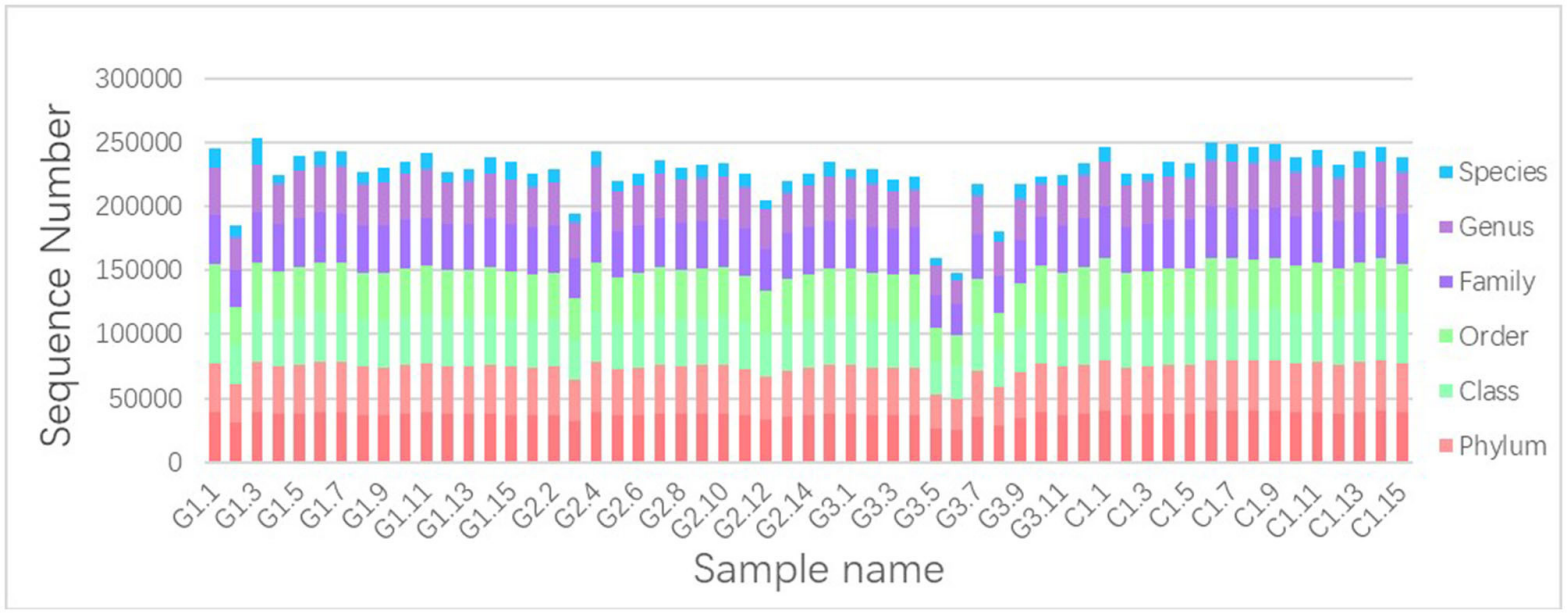
Number of gene sequences in each sample. The abscissa is the sample name; the ordinate indicates the number of sequences that are annotated to that level; the top-down color order of the histogram corresponds to the legend's color order on the right.

We used Illumina high-throughput sequencing to perform multiple pyrosequencing of the V4 hypervariable region of the 16S rRNA gene to characterize the bacterial lineage present in the fecal microbiota of all sample (20). We generated a data set consisting of 1,868,876 filtered 16S rRNA gene sequences, and the mean (±SE) of each sample was 32787.30 ± 3686.54 sequences (Figure 1, Table 1). According to the statistics of the patients' organ involvement (Table 2), children with IgA vasculitis with gastrointestinal damage have a higher rate of kidney damage. They also have fewer Sequence numbers at each classification level.

**Table 1:** Number of gene sequences in each sample (Figure 1 data supplement form).

**Sample_Name**	**Kingdom**	**Phylum**	**Class**	**Order**	**Family**	**Genus**	**Species**
GZ1.1	38750	38750	38750	38683	38674	37382	15081
GZ1.2	30618	30618	30618	29485	29188	25553	9188
GZ1.3	39208	39207	39207	39079	39044	36591	21536
GZ1.4	37312	37312	37307	37120	36808	32241	6660
GZ1.5	38226	38226	38226	38123	37970	37213	11905
GZ1.6	39113	39113	39113	39049	39007	36545	10880
GZ1.7	39056	39056	39056	38944	38756	36890	11763
GZ1.8	37230	37230	37230	36844	36432	32224	9256
GZ1.9	37154	37154	37154	37068	36225	33579	11828
GZ1.10	38021	38021	38021	37944	37563	35772	10206
GZ1.11	38442	38442	38442	38153	38009	37622	12397
GZ1.12	37649	37649	37649	37547	36334	31685	8892
GZ1.13	37578	37578	37578	37189	36892	32905	9974
GZ1.14	38234	38234	38234	38170	38064	34947	12990
GZ1.15	37266	37266	37266	37183	37125	35579	13924
GZ2.1	36875	36875	36875	36622	36415	31735	10300
GZ2.2	37259	37259	37259	36763	36730	33678	10702
GZ2.3	32054	32054	32054	31914	31891	27159	7004
GZ2.4	39255	39255	39255	38982	38965	36216	11086
GZ2.5	36221	36221	36221	36066	35993	30795	8809
GZ2.6	37044	37044	37044	36841	36782	31268	9921
GZ2.7	38290	38290	38290	38197	38112	34597	10246
GZ2.8	37556	37556	37556	37336	37285	33270	9654
GZ2.9	37923	37923	37923	37685	37601	33218	10132
GZ2.10	38157	38157	38157	37901	37834	33265	9877
GZ2.11	36583	36583	36583	36440	36402	33254	10058
GZ2.12	33441	33441	33441	33315	33274	30596	7245
GZ2.13	35882	35882	35882	35636	35581	31633	10042
GZ2.14	36827	36827	36827	36685	36622	32869	9091
GZ2.15	37916	37916	37916	37696	37623	34572	11092
GZ3.1	38046	38046	38043	37766	37566	33224	7123
GZ3.2	36949	36949	36949	36784	36774	33035	12375
GZ3.3	36672	36672	36672	36326	36178	29764	8843
GZ3.4	36864	36864	36864	36474	36430	29781	9641
GZ3.5	26228	26228	26228	26149	26117	22780	6333
GZ3.6	24884	24883	24883	24643	24506	18193	6465
GZ3.7	35837	35837	35837	35563	35548	29747	9215
GZ3.8	29087	29086	29086	28988	28966	26869	8624
GZ3.9	34956	34956	34956	34622	34619	31608	11695
GZ3.10	38453	38453	38453	38261	38250	25418	6603
GZ3.11	37226	37226	37226	36937	36900	31096	8289
GZ3.12	38321	38321	38321	38073	38002	33145	9890
CON1.1	40063	40063	40063	39817	39794	35453	11511
CON1.2	37114	37114	37114	36518	36492	32451	8837
CON1.3	37692	37692	37692	36469	36383	33774	6184
CON1.4	38093	38093	38093	37660	37615	33543	11617
CON1.5	38059	38059	38059	37651	37606	32777	12003
CON1.6	40082	40082	40082	39799	39745	35892	14623
CON1.7	40055	40055	40055	39592	39550	36278	13465
CON1.8	39847	39847	39847	39461	39405	35112	12976
CON1.9	39926	39926	39926	39540	39496	36882	13596
CON1.10	38562	38562	38562	38130	38062	34557	11920
CON1.11	39220	39220	39220	38947	38902	35711	12703
CON1.12	37928	37928	37928	37497	37442	33596	10329
CON1.13	39242	39242	39242	38918	38854	34659	13526
CON1.14	40023	40023	40023	39759	39690	35433	12117
CON1.15	38927	38927	38927	38641	38572	33245	11239

**Table 2:** Statistical table of organ involvement in children with IgA vasculitis.

	**Skin**	**Joints**	**Digestive tract**	**Kidney**
G1.1	+	-	-	-
G1.2	+	-	-	-
G1.3	+	-	-	-
G1.4	+	-	-	-
G1.5	+	-	-	-
G1.6	+	-	-	-
G1.7	+	-	-	-
G1.8	+	-	-	-
G1.9	+	-	-	-
G1.10	+	-	-	-
G1.11	+	-	-	-
G1.12	+	-	-	-
G1.13	+	-	-	-
G1.14	+	-	-	-
G1.15	+	-	-	-
G2.1	+	+	-	-
G2.2	+	+	-	-
G2.3^*^	+	+	-	+
G2.4	+	+	-	-
G2.5^*^	+	-	-	+
G2.6^*^	+	-	-	+
G2.7	+	+	-	-
G2.8	+	+	-	-
G2.9	+	+	-	-
G2.10	+	+	-	-
G2.11	+	+	-	-
G2.12^*^	+	+	-	+
G2.13	+	+	-	-
G2.14	+	+	-	-
G2.15	+	+	-	-
G3.18	+	-	+	-
G3.2	+	+	+	-
G3.3^*^	+	-	+	+
G3.4^*^	+	-	+	+
G3.5^*^	+	+	+	+
G3.6^*^	+	+	+	+
G3.7	+	+	+	-
G3.8^*^	+	+	+	+
G3.9^*^	+	+	+	+
G3.10^*^	+	+	+	+
G3.11	+	+	+	-
G3.12	+	+	+	-

* *Children with IgA vasculitis involving kidney damage*. *“+” represents the presence of symptoms involving the organ, and “−” represents no symptoms which affect the organ*.

**Figure 2:**
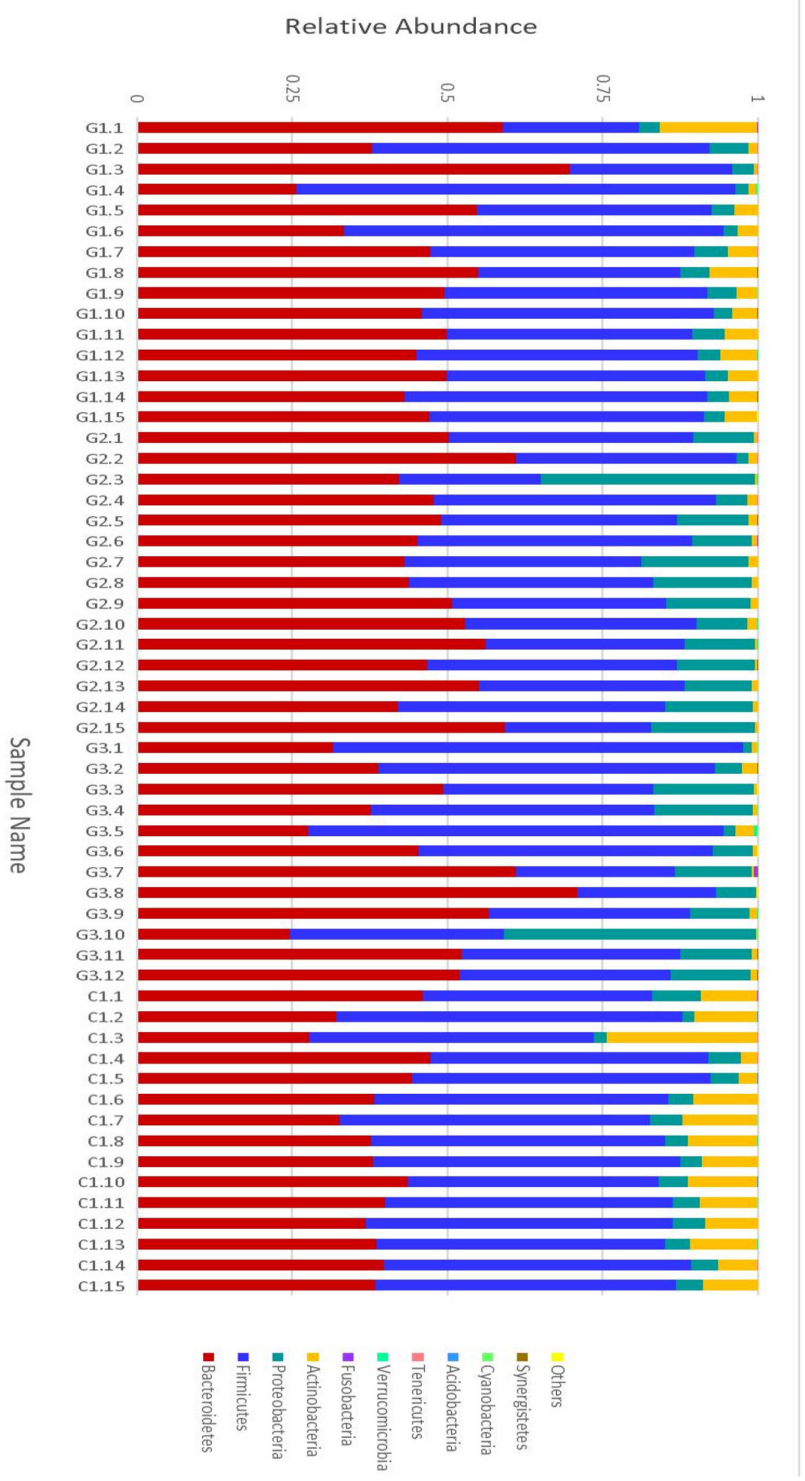
Relative abundance of the intestinal flora at the family level in children. The abscissa is the sample name; the ordinate indicates the relative abundance; “Others” represents the sum of the relative abundance of all the orders of the 10 largest phyla with the highest relative abundance (The maximum relative abundance of a phylum in all samples).

**Figure 3:**
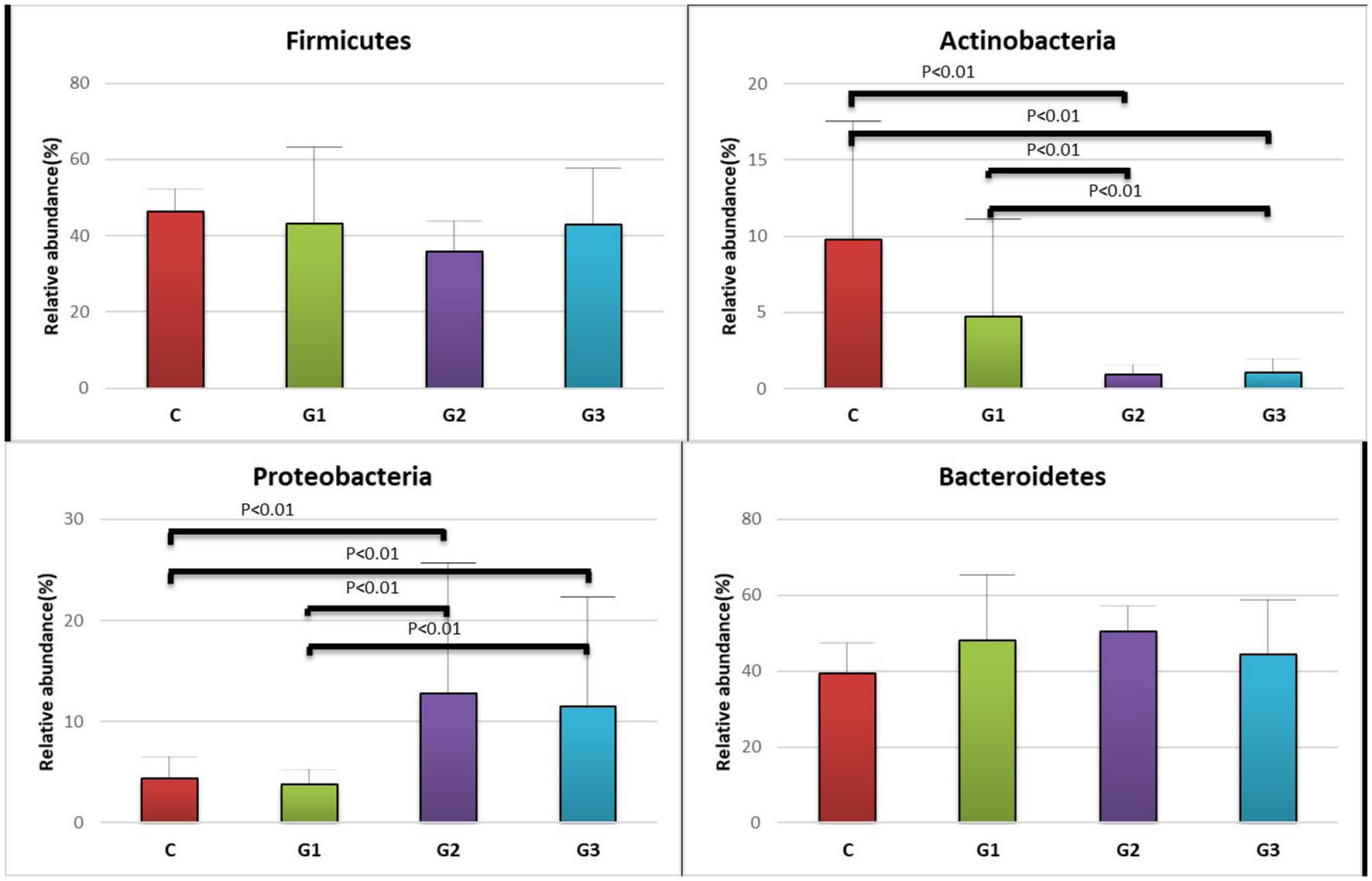
Relative abundance of the intestinal flora at the family level in the four groups of children (Firmicutes, Actinobacteria, Proteobacteria, Bacteroidetes). The abscissa is the group name; the ordinate indicates the percentage of abundance of a particular intestinal flora; the column color corresponds to the legend color on the right. **(A)** Relative abundance (%) of Firmicutes in the intestinal flora of the four groups of children. **(B)** Relative abundance (%) of Actinobacteria in the intestinal flora of the four groups of children. **(C)** Relative abundance (%) of Proteobacteria, in the intestinal flora of the four groups of children. **(D)** Relative abundance (%) of Bacteroidetes, in the intestinal flora of the four groups of children.

More than 10 bacterial phyla were detected in all samples, and more than 92.6% of all samples were found to belong to the four most-populated bacterial phyla. They were Bacteroidetes, Firmicutes, Proteobacteria and Actinobacteria (Figure 2, Table 1). The Relative abundance of predominant intestinal flora in children with IgA vasculitis and healthy controls has been counted (Table 3). Bacteroidetes and Firmicutes were the two most abundant bacteria in all samples. The relative abundances of Firmicutes did not significantly differ in G1, G2, G3 and C groups (43.5, 36.3, 41.3, and 45.3%, respectively, Figure 3A). Similarly, the relative abundances of Bacteroidetes also did not significantly differ by study groups (47.2, 49.5, 45.3, and 38.7%, respectively, Figure 3D). In addition to Bacteroidetes and Firmicutes, the microbial population with a high proportion of abundance in the study population is Proteobacteria and Actinobacteria. The proportion of Actinobacteria in G1, G2, G3 and C groups were 4.8, 1.0, 1.0, and 9.8% (Figure 3B). And the proportion of Proteobacteria were 3.8 and 12.8%, 11.5 and 4.3% (Figure 3C), respectively. The relative abundance of Proteobacteria in the G2 and G3 groups was much higher than that in the G1 and C groups (*P* < 0.001), while the relative abundance of Actinobacteria was significantly lower than that in the G1 and C groups (*P* < 0.001). In addition, the relative abundances of Actinobacteria in G1 and C groups were also significantly different. The relative abundance Actinobacteria in G1 group was lower than that in C group (*P* < 0.001) (Table 1).

**Table 3:** Relative abundance of predominant intestinal flora in children with IgA vasculitis and healthy controls.

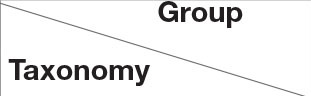	**GZ1**	**GZ2**	**GZ3**	**CON**
Bacteroidetes	47.2 ± 10.5	49.5 ± 6.2	45.3 ± 13.9^†^	38.7 ± 5.3
Firmicutes	43.5 ± 12.4	36.3 ± 6.6	41.3 ± 14.8	45.3 ± 7.4
Proteobacteria	3.8 ± 1.2	12.8 ± 7.3***	11.5 ± 10.3***	4.3 ± 1.4
Actinobacteria	4.8 ± 3.6***	1.0 ± 0.5***	1.0 ± 0.9***	9.8 ± 4.9

Results shown are the mean percent of total ± SD. Asterisks indicate comparison with CON group, * <0.05, ** <0.01, *** <0.001; Crosses indicate comparison of IgA vasculitis groups without intestinal involvement (GZ1 and GZ2) vs. IgA vasculitis with intestinal involvement (GZ3),^†^ <0.05,^††^ <0.01,^†††^< 0.001.

### 16S rRNA Genetic Survey Distinguishes Children With Different Types of IgA Vasculitis

**Figure 4:**
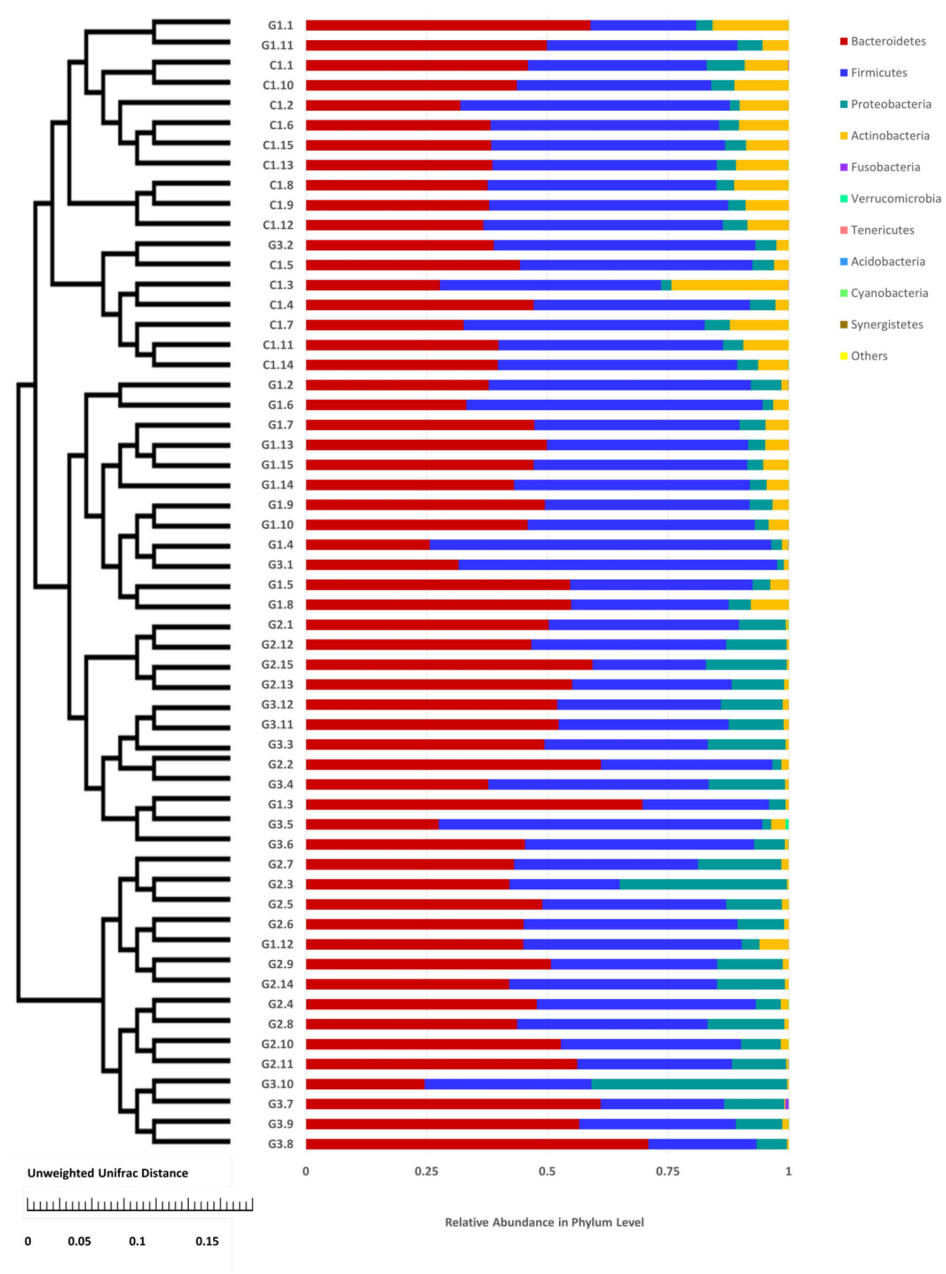
Cluster of relative abundance of the intestinal flora at the phylum level in each sample. The left side is the UPGMA cluster tree structure, on the right is the sample at the phylum level of the relative abundance of specie distribution map.

**Figure 5:**
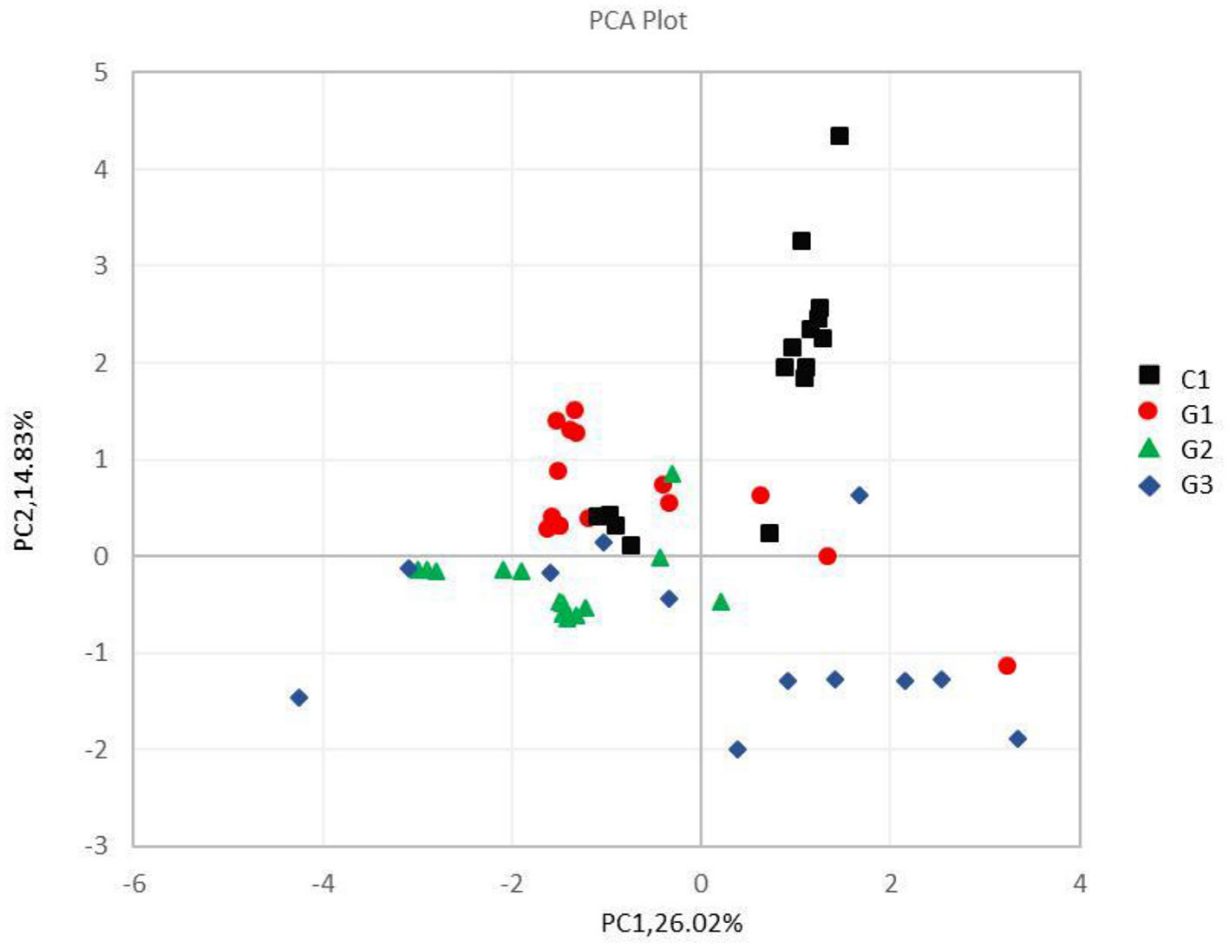
Principal component analysis of different samples at the phylum level. The abscissa and the ordinate represent two principal components, respectively, and the percentage indicates the contribution of the principal component to the sample difference; the same group of samples is represented by the same color dot.

Complete linkage hierarchical clustering divides most of the samples in the G1, G2, G3, and C groups into different clusters (Figures 4, 5). Of course, some of these samples are separated in other groups. However, this individual phenomenon has no significant impact on the analysis and judgment of the resulting trend. Notably, the samples were divided into several clusters. The resemblance between these clusters was low, which indicates that the intestinal microbiotas in these groups are different from one another. We have found a wealth of Bacteroides (Figure 2), in which the Enterobacteriaceae family has the highest relative abundance, in all four groups. We also observed abundant Firmicutes; it was found in all subjects (Figure 2) and this phylum was previously thought to be strictly related to children with other autoimmune diseases (21). The results showed a clear association between different clinical symptoms of IgA vasculitis and gut microbial composition.

### Microbial Richness and Biodiversity

**Figure 6:**
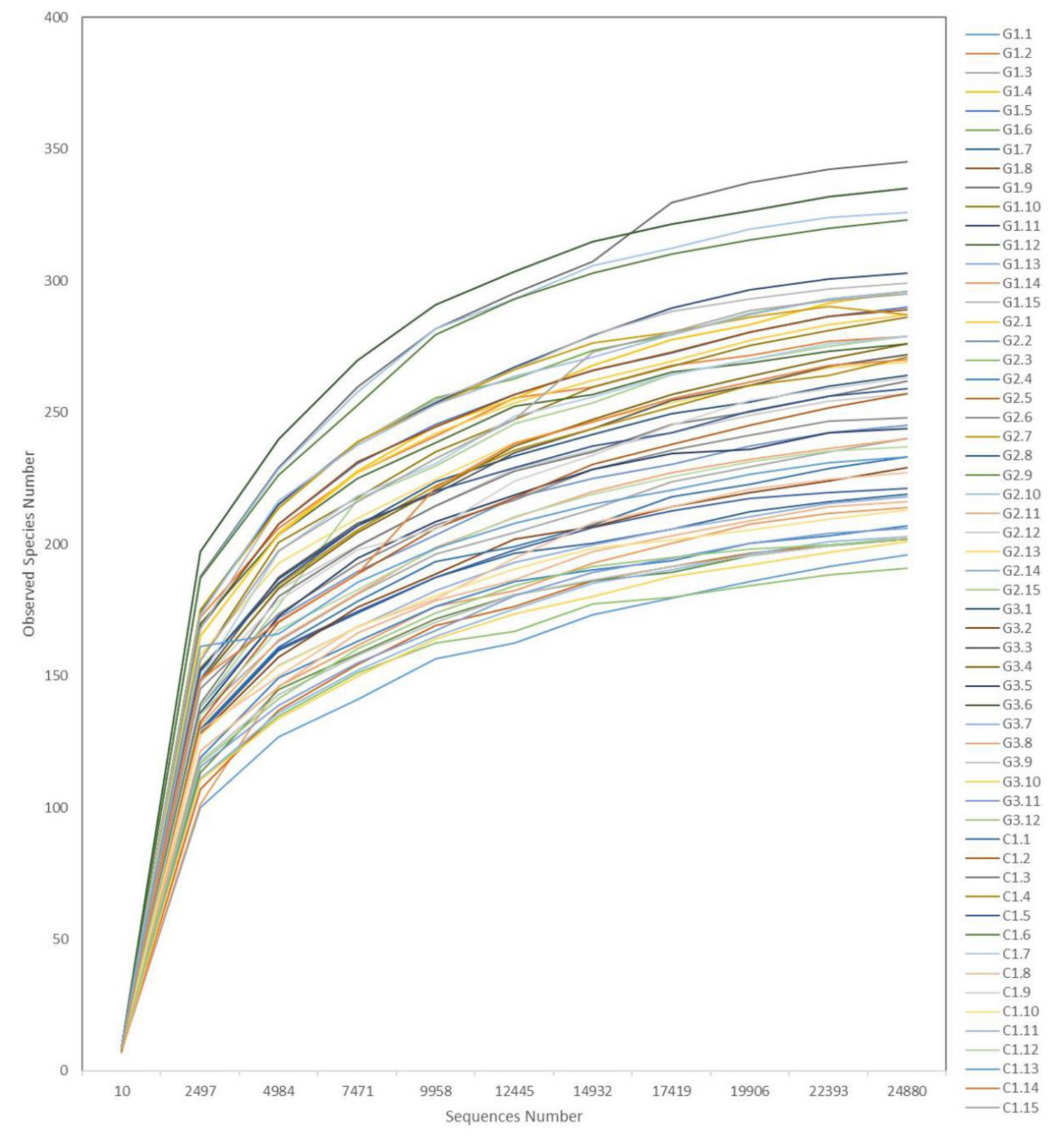
Representative rarefaction curves. The abscissa is the number of valid sequences per sample, the ordinate is the number of operational taxonomic units (OTUs) species observed. Each curve represents a different sample.

Rarefaction curves (Figure 6) showed a plateau in the number of new observed OTUs when the number of sequences evaluated for each sample was >10,000. Thus, evaluation of >24,000 sequences for each sample ensured that the sequencing data volume was sufficient to reflect the information of most microorganisms present in each sample.

## Discussion

The major findings of this study are (1) The abundance of intestinal microbes in children with IgA vasculitis is lower than in healthy children, (2) children with IgA vasculitis who present with different organ involvement show distinct patterns of intestinal microbiota, (3) Proteobacteria are increased and Actinobacteria are decreased in patients with IgA vasculitis who present with joint or intestinal symptoms compared to patients with only skin symptoms, (4) similar to other autoimmune diseases, Bacteroidetes, Firmicutes, Proteobacteria and Actinobacteria are the four most abundant bacteria in the intestinal flora of children with IgA vasculitis.

Human intestinal flora are typically composed of about 30 genera and more than 500 kinds of bacteria, which together play an important role in promoting food digestion and synthesizing protein, vitamins, and other nutrients, as well as in resisting the invasion of foreign pathogens and enhancing the immune system (22). The types, numbers, and distribution of normal flora vary due to differences in factors, such as age, race, dietary structure, and lifestyle. And both the structure and number of intestinal flora can be changed by a variety of diseases (23). Cumulatively, human gut bacteria contain more than three million genes, meaning 100 times more than human hosts (24). They have a variety of metabolic functions such as dietary component metabolism, drug detoxification, vitamins and essential amino acids synthesis (25). Different types of bacteria have different abilities in performing a certain metabolic function, that is, a specific microbiota is more effective or less effective in performing certain functions than other microbial groups (26). In recent years, increasingly more studies on perinatal and neonatal intestinal microbes have found that gestational age, delivery mode, and feeding methods each affect the composition of neonatal intestinal flora (27), and probiotics can effectively prevent the occurrence of enterocolitis (28). A variety of autoimmune diseases, such as multiple sclerosis (29) and autoimmune encephalitis (30), have also been confirmed as being impacted by intestinal microbial regulation.

The association of gut microbiota composition with IgA vasculitis and different clinical presentations of IgA vasculitis has not previously been studied. Our study used Illumina sequencing technology to study the composition of the intestinal flora of children with different clinical symptoms of IgA vasculitis and found that the microbial diversity in the feces of patients was high. The results show that more than 92.3% of genes in all samples belong to Bacteroidetes, Firmicutes, Proteobacteria, or Actinobacteria, which is in agreement with a previous study describing these phyla as composing the majority of gut microbiota found in children with other autoimmune diseases. The previous research also reported that the dominant human intestinal flora mainly belonged to the phyla Bacteroidetes and Firmicutes (31, 32), and our study found the same results.

Our study showed that the relative abundance of the gut microbiota in the CON group was higher than that in the IgA vasculitis group. This is consistent with the related research of other researchers (33). By comparing the microbial richness, we found that the abundance of intestinal bacteria was significantly lower in children with IgA vasculitis than in healthy children, and it was higher in children with other clinical manifestations of IgA vasculitis than in those with only purpura. This difference is more pronounced in children with IgA vasculitis who are involved in the gastrointestinal tract, especially in children with IgA vasculitis who have both gastrointestinal and renal damage. Previous work has applied metabolomics to querying the function of gut microbe communities in other autoimmune diseases. Studies of intestinal flora in patients with rheumatoid arthritis have shown that ion diversity in feces in patients with early rheumatoid arthritis (ERA) is significantly lower than in healthy controls (34). At the same time, similar findings are also reflected in other connective tissue diseases. Compared with healthy controls, patients with Sjögren's syndrome have lower metabolic diversity reported in saliva samples (35), and patients with psoriatic arthritis (36) and inflammatory bowel disease (IBD) (37) have lower intestinal microbiota diversity. In addition, studies of dextran sulfate-induced colitis mice have also shown that fecal insulin autoantibodies (IAA) levels are low (38).

Ather et al. reported that segmented Filamentous bacteria can promote the differentiation of Th17, resulting in an increased immune response (39). In our study, although Firmicutes tended to be represented slightly more in the intestinal flora of healthy children than in that of children with IgA vasculitis, these differences were not significant. However, we found significant differences in the abundance of Proteobacteria between healthy children and those with IgA vasculitis. Proteobacteria was not only more represented in the IgA vasculitis groups than in the CON group, but it was also found more frequently in children with IgA vasculitis who had mixed clinical syndromes than in those who had only skin injury. These results suggest that Proteobacteria may be another important group of bacteria that has an important effect on the regulation of immune disorders and on the remission of the associated disease. In contrast, the abundance of Actinobacteria was significantly lower in children with IgA vasculitis than in healthy controls, especially in those with mixed clinical syndromes. However, Bacteroides fragilis, which has been previously implicated in inducing the development of Tregs to suppress immune response (40), did not show the same trends. Therefore, further research is needed to determine whether or not Actinobacteria can affect the activity of immune cells and effectors.

In summary, the dominant human intestinal flora mainly belonged to the phyla Bacteroidetes and Firmicutes. As with several other autoimmune diseases, the incidence and progression of IgA vasculitis is also affected by the composition of gut microbiota. The number and abundance of intestinal microbes in children with IgA vasculitis are low, especially in children with multiple organs involvement including both gastrointestinal and renal damage. Proteobacteria and Actinobacteria may play an important role in the pathogenesis and prognosis of IgA vasculitis. The relative abundance of Actinobacteria was significantly reduced in all affected groups and was lower in children with multiple organ damage. Therefore, Actinobacteria may also be associated with the severity of children with IgA vasculitis. Treatment with active supplementation of this flora in the early stages of the disease may reduce organ damage and reduce the severity of IgA vasculitis. This study also has certain limitations such as small sample size. And no flora intervention therapy had been given because there was currently no suitable targeted flora. Further relevant supplements will be made in the follow-up research. In general, this research provided a new way to describe the pathogenesis of IgA vasculitis and may aid in the future development of microorganism treatments.

## Conclusions

The abundance of intestinal microbes in children with IgA vasculitis is lower than in normal children. Bacteroidetes, Firmicutes, Proteobacteria and Actinobacteria are the four most abundant bacteria in the intestinal flora of children. Proteobacteria and Actinobacteria are associated with organ involvement in IgA vasculitis.

## Data Availability Statement

The datasets presented in these studies can be found in online repositories. The names of the repository and accession number can be found below: NCBI Sequence Read Archive, accession SAMN22814273.

## Ethics Statement

The studies involving human participants were reviewed and approved by Ethics Committee on Scientific Research of Shandong University Qilu Hospital. Written informed consent was obtained from the individual(s), and minor(s)' legal guardian/next of kin, for the publication of any potentially identifiable images or data included in this article.

## Author Contributions

ML: writing—original draft preparation. XW: validation and data analysis. XB and RJ: conceptualization and methodology. AZ and AF: writing—review and editing. AZ: project administration. All authors contributed to the article and approved the submitted version.

## Funding

This work was supported by Key Research and Development Program of Shandong Province (2019GSF108060), Shandong Provincial Natural Science Foundation (ZR202010220039).

## Conflict of Interest

The authors declare that the research was conducted in the absence of any commercial or financial relationships that could be construed as a potential conflict of interest.

## Publisher's Note

All claims expressed in this article are solely those of the authors and do not necessarily represent those of their affiliated organizations, or those of the publisher, the editors and the reviewers. Any product that may be evaluated in this article, or claim that may be made by its manufacturer, is not guaranteed or endorsed by the publisher.

## References

[1] Kaya Akca U Batu ED Serin O Ipek OF Aydin O Teksam O. Penile involvement of immunoglobulin a vasculitis/Henoch-Schönlein purpura. J Pediatr Urol. (2021) 17:409.e1–409.e8. 10.1016/j.jpurol.2021.01.012 33558175

[2] Borovitz Y Alfandary H Haskin O Levi S Kaz S Davidovits M. Lower prednisone dosing for steroid-sensitive nephrotic syndrome relapse: a prospective randomized pilot study. Eur J Pediatr. (2020) 179:279–83. 10.1007/s00431-019-03506-5 31728673

[3] Boutin RCT Sbihi H Dsouza M Malhotra R Petersen C Dai D. Mining the infant gut microbiota for therapeutic targets against atopic disease. Allergy. (2020) 75:2065–8. 10.1111/all.14244 32086944

[4] Leung AKC Barankin B Leong KF. Henoch-Schonlein purpura in children: an updated review. Curr Pediatr Rev. (2020) 16:265–76. 10.2174/1573396316666200508104708 32384035

[5] Dong G Zhang J Yang Z Feng X Li J Li D. The association of gut microbiota with idiopathic central precocious puberty in girls. Front Endocrinol. (2019) 10:941. 10.3389/fendo.2019.00941 32038493 PMC6987398

[6] Zambrana LE McKeen S Ibrahim H Zarei I Borresen EC Doumbia L. Rice bran supplementation modulates growth, microbiota and metabolome in weaning infants: a clinical trial in Nicaragua and Mali. Sci Rep. (2019) 9:13919. 10.1038/s41598-019-50344-4 31558739 PMC6763478

[7] Hao B Wang K Zhou Y Sui C Wang L Bai R. Label-free detecting of the compaction and decompaction of ctDNA Molecules induced by surfactants with SERS based on a nanoPAA-ZnCl2-AuLs solid substrate. ACS Omega. (2020) 5:1109–19. 10.1021/acsomega.9b03294 31984267 PMC6977030

[8] Magoc T Salzberg SL. FLASH: fast length adjustment of short reads to improve genome assemblies. Bioinformatics. (2011) 27:2957–63. 10.1093/bioinformatics/btr507 21903629 PMC3198573

[9] Edgar RC. UPARSE: highly accurate OTU sequences from microbial amplicon reads. Nat Methods. (2013) 10:996–8. 10.1038/nmeth.2604 23955772

[10] Liu W Zhang R Shu R Yu J Li H Long H. Study of the relationship between microbiome and colorectal cancer susceptibility using 16SrRNA sequencing. Biomed Res Int. (2020) 2020:7828392. 10.1155/2020/7828392 32083132 PMC7011317

[11] Caporaso JG Bittinger K Bushman FD DeSantis TZ Andersen GL Knight R. PyNAST: a flexible tool for aligning sequences to a template alignment. Bioinformatics. (2010) 26:266–7. 10.1093/bioinformatics/btp636 19914921 PMC2804299

[12] Son HJ Kim N Song CH Nam RH Choi SI Kim JS. Sex-related alterations of gut microbiota in the C57BL/6 mouse model of inflammatory bowel disease. J Cancer Prev. (2019) 24:173–82. 10.15430/JCP.2019.24.3.173 31624723 PMC6786806

[13] Geng H Shu S Dong J Li H Xu C Han Y. Association study of gut flora in Wilson's disease through high-throughput sequencing. Medicine. (2018) 97:e11743. 10.1097/MD.0000000000011743 30075590 PMC6081054

[14] Zhao MM Du SS Li QH Chen T Qiu H Wu Q. High throughput 16SrRNA gene sequencing reveals the correlation between Propionibacterium acnes and sarcoidosis. Respir Res. (2017) 18:28. 10.1186/s12931-017-0515-z 28143482 PMC5286795

[15] White JR Nagarajan N Pop M. Statistical methods for detecting differentially abundant features in clinical metagenomic samples. PLoS Comput Biol. (2009) 5:e1000352. 10.1371/journal.pcbi.1000352 19360128 PMC2661018

[16] Moreno-Arrones OM Serrano-Villar S Perez-Brocal V Saceda-Corralo D Morales-Raya C Rodrigues-Barata R. Analysis of the gut microbiota in alopecia areata: identification of bacterial biomarkers. J Eur Acad Dermatol Venereol. (2020) 34:400–5. 10.1111/jdv.15885 31419351

[17] Quagliariello A Del Chierico F Russo A Reddel S Conte G Lopetuso LR. Gut microbiota profiling and gut-brain crosstalk in children affected by pediatric acute-onset neuropsychiatric syndrome and pediatric autoimmune neuropsychiatric disorders associated with streptococcal infections. Front Microbiol. (2018) 9:675. 10.3389/fmicb.2018.00675 29686658 PMC5900790

[18] Garza-Gonzalez E Mendoza-Olazaran S Morfin-Otero R Ramirez-Fontes A Rodriguez-Zulueta P Flores-Trevino S. Intestinal microbiome changes in fecal microbiota transplant (FMT) vs FMT enriched with *Lactobacillus* in the treatment of recurrent clostridioides difficile Infection. Can J Gastroenterol Hepatol. (2019) 2019:4549298. 10.1155/2019/4549298 31976311 PMC6955117

[19] Citronberg JS Curtis KR White E Newcomb PA Newton K Atkinson C. Association of gut microbial communities with plasma lipopolysaccharide-binding protein (LBP) in premenopausal women. ISME J. (2018) 12:1631–41. 10.1038/s41396-018-0064-6 29434315 PMC6018759

[20] Bai H Jin W Guo J Ding Y Chang C Guo X. lncRNA expression reveals the potential regulatory roles in hepatocyte proliferation during rat liver regeneration. Biomed Res Int. (2019) 2019:8597953. 10.1155/2019/8597953 31828136 PMC6885160

[21] Tremlett H Fadrosh DW Faruqi AA Hart J Roalstad S Graves J. Associations between the gut microbiota and host immune markers in pediatric multiple sclerosis and controls. BMC Neurol. (2016) 16:182. 10.1186/s12883-016-0703-3 27652609 PMC5031272

[22] Postler TS Ghosh S. Understanding the holobiont: how microbial metabolites affect human health and shape the immune system. Cell Metab. (2017) 26:110–30. 10.1016/j.cmet.2017.05.008 28625867 PMC5535818

[23] Wang X Zhang L Wang Y Liu X Zhang H Liu Y. Gut microbiota dysbiosis is associated with henoch-schonlein purpura in children. Int Immunopharmacol. (2018) 58:1–8. 10.1016/j.intimp.2018.03.003 29525681

[24] Holman DB Bearson BL Allen HK Shippy DC Loving CL Kerr BJ. Chlortetracycline enhances tonsil colonization and fecal shedding of multidrug-resistant *Salmonella enterica* serovar typhimurium DT104 without major alterations to the porcine tonsillar and intestinal microbiota. Appl Environ Microbiol. (2019) 85:e02354–18. 10.1128/AEM.02354-18 30530706 PMC6365817

[25] Sakkas H Bozidis P Touzios C Kolios D Athanasiou G Athanasopoulou E. Nutritional status and the influence of the vegan diet on the gut microbiota and human health. Medicina. (2020) 56:88. 10.3390/medicina56020088 32098430 PMC7073751

[26] Zhang Y Xia G Nie X Zeng Y Chen Y Qian Y. Differences in manifestations and gut microbiota composition between patients with different henoch-schonlein purpura phenotypes. Front Cell Infect Microbiol. (2021) 11:641997. 10.3389/fcimb.2021.641997 34277463 PMC8281929

[27] Collado MC Cernada M Neu J Perez-Martinez G Gormaz M Vento M. Factors influencing gastrointestinal tract and microbiota immune interaction in preterm infants. Pediatr Res. (2015) 77:726–31. 10.1038/pr.2015.54 25760550

[28] Chang HY Chen JH Chang JH Lin HC Lin CY Peng CC. Multiple strains probiotics appear to be the most effective probiotics in the prevention of necrotizing enterocolitis and mortality: An updated meta-analysis. PLoS ONE. (2017) 12:e0171579. 10.1371/journal.pone.0171579 28182644 PMC5300201

[29] Buscarinu MC Fornasiero A Romano S Ferraldeschi M Mechelli R Renie R. The Contribution of gut barrier changes to multiple sclerosis pathophysiology. Front Immunol. (2019) 10:1916. 10.3389/fimmu.2019.01916 31555257 PMC6724505

[30] Weissert R. Adaptive immunity is the key to the understanding of autoimmune and paraneoplastic inflammatory central nervous system disorders. Front Immunol. (2017) 8:336. 10.3389/fimmu.2017.00336 28386263 PMC5362596

[31] Mushtaq N Hussain S Zhang S Yuan L Li H Ullah S. Molecular characterization of alterations in the intestinal microbiota of patients with grade 3 hypertension. Int J Mol Med. (2019) 44:513–22. 10.3892/ijmm.2019.4235 31173179 PMC6605625

[32] Bradley PH Pollard KS. Proteobacteria explain significant functional variability in the human gut microbiome. Microbiome. (2017) 5:36. 10.1186/s40168-017-0244-z 28330508 PMC5363007

[33] Cao J Wu C Wang K Hu H Duan J Zhao B. Metagenomic profiling reveals dominance of gram-positive bacteria in the gut microbiome shifts associated with immunoglobulin A vasculitis (Henoch-Schonlein Purpura). Clin Transl Immunology. (2021) 10:e1342. 10.1002/cti2.1342 34646556 PMC8499602

[34] Stoll ML Kumar R Lefkowitz EJ Cron RQ Morrow CD Barnes S. Fecal metabolomics in pediatric spondyloarthritis implicate decreased metabolic diversity and altered tryptophan metabolism as pathogenic factors. Genes Immun. (2016) 17:400–5. 10.1038/gene.2016.38 27786174 PMC5133160

[35] Kageyama G Saegusa J Irino Y Tanaka S Tsuda K Takahashi S. Metabolomics analysis of saliva from patients with primary Sjogren's syndrome. Clin Exp Immunol. (2015) 182:149–53. 10.1111/cei.12683 26201380 PMC4608504

[36] Boer CG Radjabzadeh D Medina-Gomez C Garmaeva S Schiphof D Arp P. Intestinal microbiome composition and its relation to joint pain and inflammation. Nat Commun. (2019) 10:4881. 10.1038/s41467-019-12873-4 31653850 PMC6814863

[37] Lavelle A Sokol H. Gut microbiota-derived metabolites as key actors in inflammatory bowel disease. Nat Rev Gastroenterol Hepatol. (2020) 17:223–37. 10.1038/s41575-019-0258-z 32076145

[38] Shiomi Y Nishiumi S Ooi M Hatano N Shinohara M Yoshie T. GCMS-based metabolomic study in mice with colitis induced by dextran sulfate sodium. Inflamm Bowel Dis. (2011) 17:2261–74. 10.1002/ibd.21616 21287666

[39] Yi J Jung J Han D Surh CD Lee YJ. Segmented filamentous bacteria induce divergent populations of antigen-specific CD4 T cells in the small intestine. Mol Cells. (2019) 42:228–36. 10.14348/molcells.2018.0424 30759969 PMC6449712

[40] Maerz JK Trostel C Lange A Parusel R Michaelis L Schafer A. Bacterial immunogenicity is critical for the induction of regulatory B cells in suppressing inflammatory immune responses. Front Immunol. (2019) 10:3093. 10.3389/fimmu.2019.03093 32038631 PMC6993086

